# *Notes from the Field:* Vitamin D–Deficient Rickets and Severe Hypocalcemia in Infants Fed Homemade Alkaline Diet Formula — Three States, August 2020–February 2021

**DOI:** 10.15585/mmwr.mm7033a4

**Published:** 2021-08-20

**Authors:** Diane P. Calello, Mohamed Jefri, Melissa Yu, Joseph Zarraga, David Bergamo, Richard Hamilton

**Affiliations:** ^1^Department of Emergency Medicine, Rutgers New Jersey Medical School, Newark, New Jersey; ^2^New Jersey Poison Information and Education System, Newark, New Jersey; ^3^Crozer-Chester Medical Center, Upland, Pennsylvania; ^4^AI DuPont Hospital for Children, Wilmington, Delaware; ^5^Department of Emergency Medicine, Drexel University College of Medicine, Philadelphia, Pennsylvania.

During August 2020–February 2021, three infants were treated in separate emergency departments in New Jersey, Pennsylvania, and Delaware for symptoms related to consumption of a nutritionally deficient homemade formula based on alkaline diet recipes, with resultant severe hypocalcemia and vitamin D–deficient rickets. Homemade infant formulas and vegan diets might be deficient in essential vitamins and nutrients as has been reported for other formulas (*1*,*2*).

**Case 1**. On January 29, 2021, a male infant aged 4 months experienced respiratory distress at home and became unresponsive. Emergency medical services found the infant to be pale, lethargic, tachycardic, and hypoxemic (oxygen saturation = 80% [normal ≥95%]), and transported him to the hospital, where he experienced several episodes of bradycardia and cardiac arrest despite emergency endotracheal intubation and mechanical ventilation, insertion of a central venous catheter, fluid replacement, and high-dose intravenous calcium. After the infant was successfully resuscitated, computed tomography and magnetic resonance imaging scans indicated that the infant had diffuse hypoxic brain injury. Laboratory evaluation revealed profound electrolyte abnormalities (anion gap acidosis pH: 6.67 [normal = 7.35–7.45], lactate: 8.3 mmol/L [normal = 0.22–2.98 mmol/L], serum sodium: 164 mEq/L [normal = 135–145 mEq/L], potassium: 6.9 mEq/L [normal = 3.5–5.5 mEq/L], and calcium: 4.0 mg/dL [normal = 8.8–10.8 mg/dL]). Radiographs showed diffuse bone demineralization with flaring and irregularities of long-bone metaphyses consistent with rickets. The child had been fed a homemade formula of sea moss (an intended iodine source), hemp seeds, and coconut water for approximately 1 month.

**Case 2**. On January 26, 2021, a male infant aged 5 months was treated in an emergency department after experiencing an episode of extremity stiffening, cyanosis, and brief apnea. Laboratory evaluation revealed that serum calcium was 4.5 mg/dL (normal = 8.8–10.8 mg/dL) with elevated alkaline phosphatase and lactate. Radiographs showed diffuse demineralization with fraying metaphyseal contours consistent with rickets. His parents reported transitioning him at age 3 months to a homemade formula made of coconut water, hemp seed hearts, dates, sea moss gel, and alkaline water. He received high-dose intravenous calcium and magnesium and was discharged home after being placed on a diet of commercial infant formula.

**Case 3**. On August 7, 2020, a male infant aged 9 months was evaluated after 5 days of irritability. Physical examination revealed weight and length below the third percentile, frontal bossing (prominent, protruding forehead), decreased tone (inability to sit without assistance), and gross and fine motor delays. Laboratory evaluation showed severe hypocalcemia, no detectable vitamin D, and a thyroid stimulating hormone level of 94,600 mU/L (normal = 0.5–5 mU/L). Long-bone radiographs demonstrated frayed metaphyses and tibial bowing. The patient received diagnoses of rickets and iodine deficiency. His parents reported feeding him homemade formula on an alkaline vegan diet consisting of coconut milk, dates, and sea moss, although the sea moss had been discontinued several months earlier. He was treated with iodine and calcium supplementation and was discharged to a long-term care facility. 

Each of these infants had been fed a homemade formula, reported by their parents as the alkaline diet. Recipes associated with this diet, several variations of which can be found online, show it lacks essential vitamins and micronutrients such as vitamin D, calcium, and iodine. CDC and the Food and Drug Administration have issued warnings about the use of homemade infant formula.*^,^^†^ These three cases highlight the potential for grave consequences (*1*,*2*). Parents should be cautioned to avoid this inappropriate substitute for breast milk or commercial infant formula that can cause hypovitaminosis D, hypocalcemic cardiorespiratory failure, and hypothyroidism, resulting in lasting harm and possibly death.

Human breast milk and commercial infant formula contain vitamins and micronutrients essential for growth and development (*3*). Infants fed an alternative diet can develop severe deficiencies and experience long-lasting developmental consequences. The Food and Drug Administration has advised parents and caregivers not to feed homemade formulas to infants, and guidance on choosing an infant formula is available from CDC.

## Footnotes

*
https://www.fda.gov/food/alerts-advisories-safety-information/fda-advises-parents-and-caregivers-not-make-or-feed-homemade-infant-formula-infants

†
https://www.cdc.gov/nutrition/InfantandToddlerNutrition/formula-feeding/choosing-an-infant-formula.html
